# Trends in Suicide Mortality in South Africa, 1997 to 2016

**DOI:** 10.3390/ijerph17061850

**Published:** 2020-03-12

**Authors:** Tahira Kootbodien, Nisha Naicker, Kerry S. Wilson, Raj Ramesar, Leslie London

**Affiliations:** 1 National Institute for Occupational Health, National Health Laboratory Services, Constitution Hill, Johannesburg 2001, South Africa; 2 School of Public Health, Faculty of Health Sciences, University of Witwatersrand, Johannesburg 2193, South Africa; 3 Division of Human Genetics, University of Cape Town, Cape Town 7925, South Africa; 4 School of Public Health and Family Medicine, University of Cape Town, Cape Town 7925, South Africa

**Keywords:** suicide mortality, joinpoint regression analysis, trends, years of potential life lost, suicide rates

## Abstract

Suicide rates worldwide are declining; however, less is known about the patterns and trends in mortality from suicide in sub-Saharan Africa. This study evaluates trends in suicide rates and years of potential life lost from death registration data in South Africa from 1997 to 2016. Suicide (X60–X84 and Y87) was coded using the 10th Revision of the International Classification of Diseases (ICD-10). Changes in mortality rate trends were analysed using joinpoint regression analysis. The 20-year study examines 8573 suicides in South Africa, comprising 0.1% of all deaths involving persons 15 years and older. Rates of suicide per 100,000 population were 2.07 in men and 0.49 in women. Joinpoint regression analyses showed that, while the overall mortality rate for male suicides remained stable, mortality rates due to hanging and poisoning increased by 3.9% and 3.5% per year, respectively. Female suicide mortality rates increased by 12.6% from 1997 to 2004 before stabilising; while rates due to hanging increased by 3.0% per year. The average annual YPLL due to suicide was 9559 in men and 2612 in women. The results show that suicide contributes substantially to premature death and demonstrates the need for targeted interventions, especially among young men in South Africa.

## 1. Introduction

Suicide contributed approximately 817,000 deaths to global mortality in 2016, accounting for 1.5% of the total deaths in the world [1]. Findings from the Global Burden of Disease Study 2016 showed that, while an increase in the absolute number of suicide deaths worldwide was observed from 1990 to 2016, there was a significant decrease in the global age-standardised suicide mortality rate (ASMR) by a third, from 16.6 to 11.2 deaths per 100,000 population [1]. It is estimated that approximately 80% of all suicides occur in low- and middle-income countries [2]. In 2016, southern sub-Saharan Africa had the third-highest regional suicide mortality rate (ASMR 16.3 per 100,000) in the world, with approximately 11,000 suicide deaths contributing to the burden of disease [1].

Suicide is a complex behaviour consisting of an interplay between many risks and protective factors impacting at individual, family, community and societal levels [3]. Suicide varies with sex and age-groups across different countries and regions. Suicide mortality is higher among men than women in general, as women report more suicidal thoughts and men are more likely to die by suicide [4]. Suicide rates in Africa are at least three times higher in men than women [5] and approximately five times higher in South African men compared to women [6], although the distribution varies across population groups and cities [7]. Globally, suicide mortality is higher among younger adults and adults later in life, whereas in sub-Saharan Africa, the suicide mortality rate increases with age [8]. The choice of suicide method varies by socioeconomic levels of countries [9], social, religious and cultural beliefs, and availability and access to a particular method [10]. Variability on the prevalent methods of suicide (hanging, use of firearms or poisoning) has been reported across African countries [5], with hanging and poisoning reported as the most common methods used in South Africa [11,12].

Monitoring of suicide mortality data can be used as a surveillance tool to identify the mental health needs of a population. Moreover, it can inform prevention efforts by monitoring progress in the implementation of programmes to reduce the morbidity and mortality associated with mental illness [13]. However, for monitoring to be optimal, mortality data needs to be accurate, complete and timely [14]. Subsequently, efforts have been made to improve the quality of mortality data in South Africa [15]. Within this context, this study aims to analyse trends in suicide mortality and patterns in the choice of suicide methods to identify populations at risk in South Africa. 

## 2. Materials and Methods 

### 2.1. Data Sources and Data Management

We obtained the underlying cause of death data based on death certificate information reported to Statistics South Africa from the Department of Home Affairs for each year from 1997 to 2016. Data from the observation period were derived from statistics published on the Statistics South Africa website and are available online [16]. Suicide was defined as a death resulting from intentional self-harm according to the World Health Organisation (WHO) International Statistical Classification of Diseases and Related Health Problems 10th revision (ICD-10), which uses codes X60 to X84 and Y87 to identify this outcome. Methods of suicide were classified into hanging (X70), drowning (X71), self-poisoning (X60–X69), firearm discharge and explosive material (X71–X75), blunt or sharp objects (X78–X79), fire, heat and hot substances (X76–X77), jumping from a high place or moving object (X80–X81), crashing of motor vehicle (X82) and unspecified means (X83–X84). Ill-defined or unknown causes of death (R99) was 11.6% in total and ranged from 3.1% to 6.5% annually, from 1997 to 2016.

Occupation groups were categorised according to the South African Standard Classification of Occupations (SASCO): managers, professionals, technicians, clerks, service workers and armed forces personnel, skilled agricultural workers, craft and related trade workers, plant and machine operators, elementary workers and unspecified occupations or persons not economically active [17]. Elementary workers included occupations such as cleaners, agricultural and forestry labourers or farm workers and refuse workers.

### 2.2. Statistical Analysis

We used mid-year population estimates to calculate age-standardised suicide mortality rates (ASMR) by sex for all years, except for 1997 to 2001, where mid-year population by age group was not available. We used the 1996 South African census data to calculate ASMRs for 1997 to 2000, and the 2001 census data was used for that year. The rates were standardised to the WHO standard population [18]. Individuals for whom sex or age was unspecified or unknown were not included in the analyses. 

As ASMRs are influenced by deaths in older populations and the distribution of suicide mortality rates are high among younger adults as well as older populations [8], we calculated years of potential life lost (YPLL) as a measure of premature mortality that estimates the average time a person would have lived if death had not occurred prematurely [19]. The YPLL method assumes that if the person has not died from suicide, they would have lived until 65 years of age. To account for the changes in the population age-structure over time, YPLL was standardised to the WHO standard population.

We used joinpoint regression analyses to identify changes in age-standardised suicide mortality trends in men and women. Joinpoint regression is a particular type of spline regression analysis [20] that explains the relationship between two variables, in which the spline segments are constraint to be linear. We analysed changes in trends in suicide mortality by sex, over 20 years, by fitting a regression line to the natural logarithm of the suicide rates, using the year of death as a regression variable. The changes in mortality rates were summarised as (increased, decreased or remained stable) annual percentage change (APC) and 95% confidence intervals. We used the Surveillance Epidemiology and End Results statistical software (Joinpoint Regression Program, Version 4.5.0.1, National Cancer Institute, Bethesda, MD, USA) to determine the minimum number of change points, called joinpoints, by applying a permutation procedure described by Kim et al. [21,22]. The selection of change points or joinpoints was determined using the grid method described by Lerman et al. [23], and the number of significant points was determined using several permutation tests, and the overall *p*-value was tested using Bonferonni correction. A significance level of 0.05 was used for the permutation test with 4499 of randomly permuted datasets.

Multiple logistic regression analysis was performed to identify socio-demographic factors associated with suicide and method of suicide. Variables with *p*-values of <0.10 in the univariate analysis were included in the multivariate analysis. The final model is presented using mortality odds ratios (MORs) and 95% confidence intervals (CI). Level of significance was set at 0.05. Data were cleaned and analysed using Microsoft Excel 2013 and STATA version 15.

## 3. Results

### 3.1. Description of the Study Population

During the 20-year observation period (1997 to 2016), there were approximately 8.9 million recorded deaths in people 15 years and older in South Africa, of which 8573 (0.1%) deaths were due to suicide. Men accounted for 78.1% (6699) of the total suicide deaths. The mean age of suicide deaths was similar among men (35 ± 14.9 years) and women (35 ± 18.2 years, *p* = 0.535). Suicide deaths were more prevalent in December (11.7%) and January (9.2%) and lowest in winter (May to July, the average prevalence of 7.2%). 

The socio-demographic characteristics of persons who died by suicide from 1997 to 2016 are summarised in Table 1. Deaths due to suicide significantly increased across five-year intervals from 1997 to 2016 (nptrend, *p* < 0.001). Men were nearly three times more likely to die by suicide than women (adjMOR = 2.68, 95% CI 2.49–2.89). Approximately 73% (6237) of all suicides occurred in men and women aged 15 to 44 years. However, the odds of dying by suicide was highest among 15- to 29-year age group (adjMOR = 8.42, 95% CI 7.31–9.70). Black African men accounted for 52% of all suicide deaths. Asian or Indian women had the lowest prevalence of suicide (0.48%). Persons with any education (primary school and above) had increased odds of dying by suicide compared with persons who had no education or having attained grade R level education. The risk of dying by suicide increased as education level increased (nptrend, *p* < 0.001). Persons who smoked five years before their death and for at least six months were more likely to die by suicide than those who did not smoke (adjMOR = 1.24, 95% CI 1.15–1.32). Among those employed, elementary occupations had the highest prevalence of suicide (35.4%), followed by service and armed forces (14.8%). Compared to technicians, elementary occupations (adjMOR = 1.69, 95% CI 1.06–2.71), skilled agricultural workers (adjMOR = 2.72, 95% CI 1.64–4.51) and service workers and armed forces personnel (adjMOR = 1.88, 95% CI 1.16–3.06) were more likely to die by suicide. While 27% of suicide deaths occurred at home, and 20% died in a hospital or emergency room, for the largest proportion of suicides, the place of suicide deaths was unknown (44%).

### 3.2. Trends in Suicide Mortality

**Figure 1:**
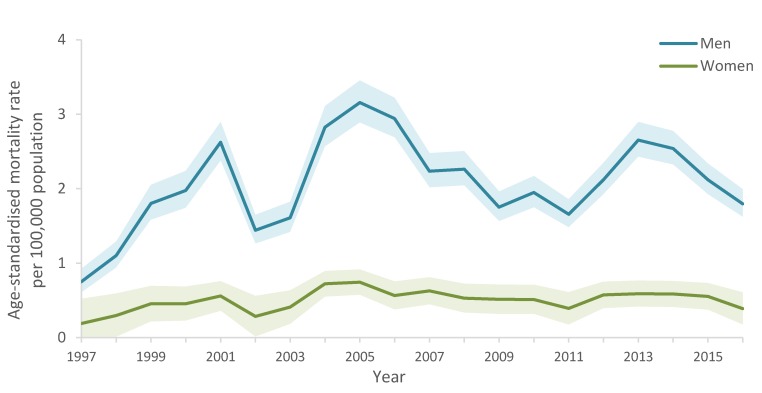
Age-standardised suicide mortality rate (95% CI bands) by sex in South Africa, 1997–2016.

From 1997 to 2016, suicide rates per 100,000 population were 2.07 in men and 0.49 in women. Men had consistently higher suicide mortality rates than women from 1997 to 2016 (Figure 1). The highest observed mortality rate for men in the time series was 3.16 deaths per 100000 population in 2005.

**Figure 2:**
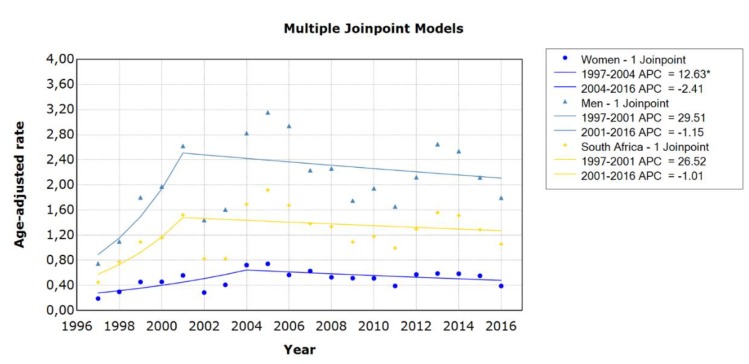
Trends in age-standardised suicide mortality rates in men and women in South Africa using joinpoint regression analysis. Data markers represent observed rates; lines represent joinpoint regression using one joinpoint; APC = annual percentage change; * indicates that the APC is significantly different from zero at the alpha = 0.05 level.

The joinpoint regression analyses identified one change point or joinpoint for men (2001) and women (2004), resulting in two linear segments or changes in the mortality rate (Figure 2). The mortality rate remained stable for men within each period identified by the model, as the increase in mortality rates from 1997 to 2001 (APC = 29.5%, 95% CI 0–67.8) and decrease in mortality rates from 2001 to 2016 (APC = −1.2%, 95% CI 4.2–2.0) were not significant. The mortality rate for women increased significantly by 12.6% (95% CI 1.5–25.0) from 1997 to 2004 and then stabilised.

### 3.3. Suicide Patterns by Age

**Figure 3:**
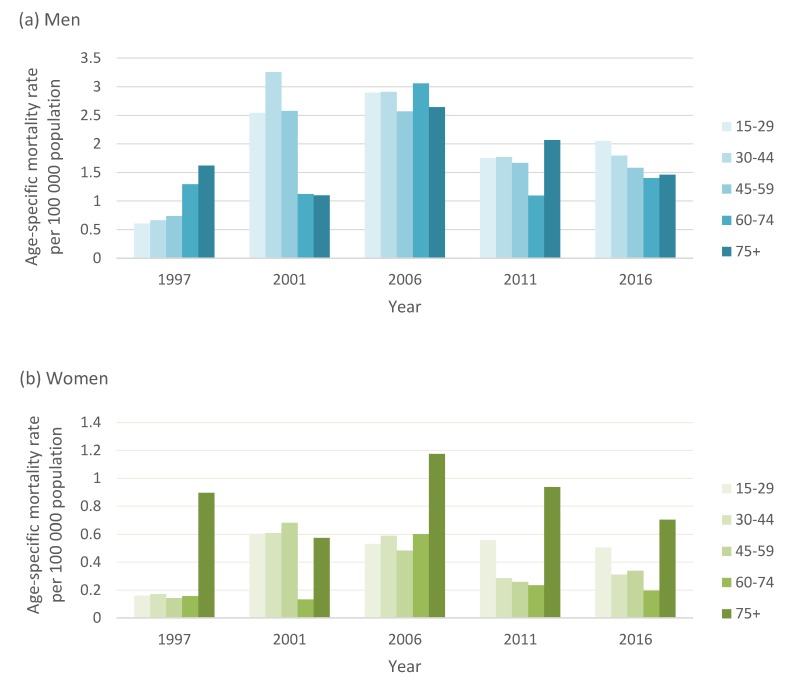
Age-specific suicide rates in men and women by broad age groups in South Africa across 5-year intervals. (**a**) Men; (**b**) Women.

Age-specific suicide rates varied substantially in men compared to women (Figure 3). In the late 1990s, suicide rates increased with age, and men 75 years and older had the highest suicide rate (1.61 deaths per 100,000). In 2006, mortality rates were high in men across all age groups. However, in 2016, men aged 15 to 29 years had the highest suicide rate (2.1 deaths per 100,000 population), followed by 30- to 44-year age group (1.8 deaths per 100,000) and gradually declined with age. In contrast, suicide mortality in women was consistently higher among those older than 75 years from 2006 onwards.

### 3.4. Trends in Suicide Methods

**Figure 4:**
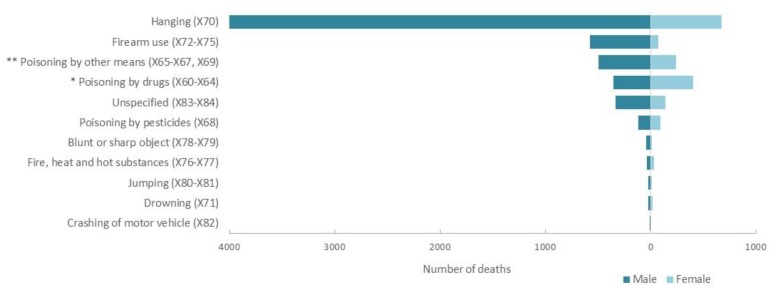
Number of deaths by method of suicide, 1997 to 2016. * Drug poisoning include medication such as analgesics, ingestion of analgesics, antiepileptic medication; narcotics and other drugs acting on the autonomic nervous system; ** poisoning by other means includes unspecified drugs, organic solvents, gases and vapours and unspecified chemicals.

In Figure 4, from 1997 to 2016, hanging was the leading method of suicide in men, accounting for 4057 (60.6%) deaths, followed by poisoning (drug poisoning, 5.3%; pesticide poisoning, 1.7%, poisoning by other means, 7.4%) and firearm use (578, 8.6%). Poisoning was the leading method of suicide in women (drug poisoning, 21.9%; pesticide poisoning, 5.0% and poisoning by other means, 12.9%), followed closely by hanging (36.1%). Deaths due to pesticide poisoning and drugs in the total population were 2.4% (208) and 8.7% (766), respectively. Approximately 6% of all suicides were not classified (X83–X84). 

**Figure 5:**
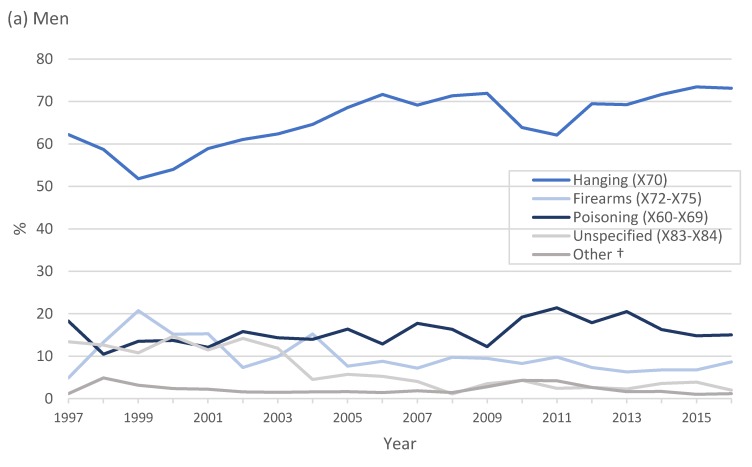

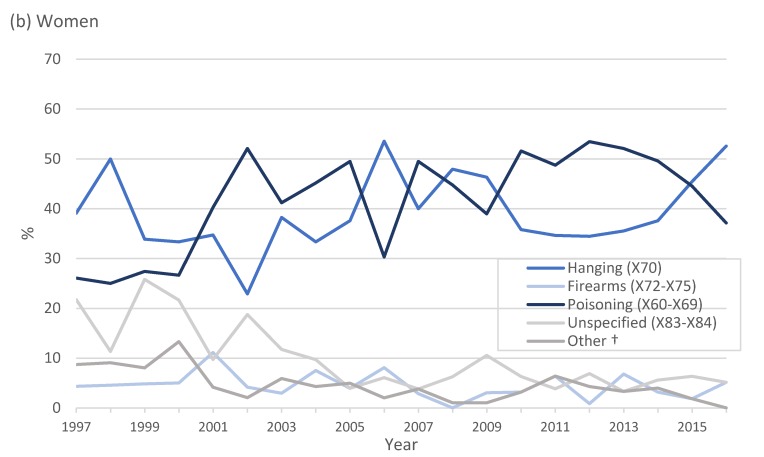
Proportion of suicide by method in (a) men and (b) women in South Africa, 1997 to 2016; † other includes intentional self-harm by drowning (X71), fire, heat and hot substances (X76–X77), blunt or sharp object (X78–X79), jumping from a high place or moving object (X81) and crashing of motor vehicle (X82). (**a**) Men; (**b**) Women.

Trends in the method of suicide varied substantially by sex (Figure 5). Hanging was the leading method of suicide in men for the study period, followed by poisoning and the use of firearms. In women, both hanging and poisoning were common methods of suicide. 

**Figure 6:**
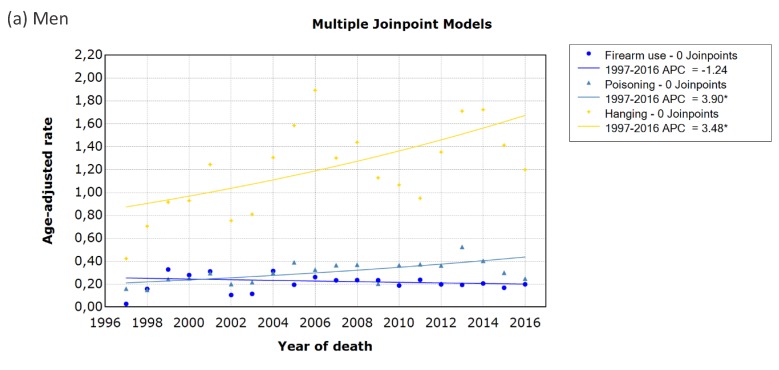

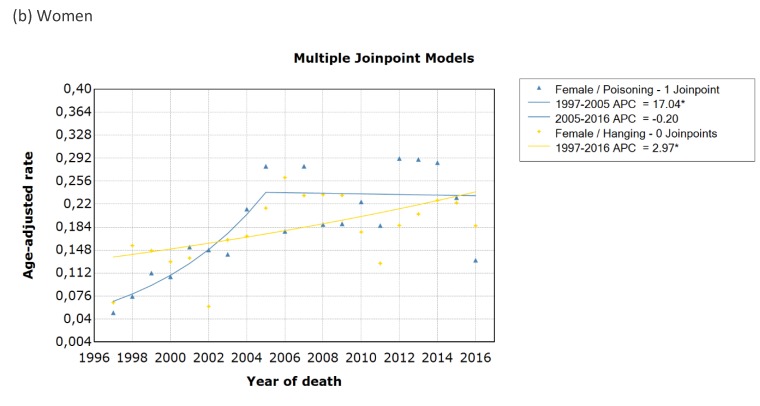
Trends in age-standardised mortality rates by the method of suicide in (a) men and (b) women in South Africa using joinpoint regression analysis. Data markers represent observed rates; lines represent joinpoint regression using no join points or one join point for poisoning in women. Joinpoint regression analysis for firearm use in women could not process records with dependent variable = 0; APC = annual percentage change; * indicates that the APC is significantly different from zero at the alpha = 0.05 level. (**a**) Men; (**b**) Women.

Joinpoint regression analysis (Figure 6) shows that deaths due hangings in men and women increased significantly by 3.5% (95% CI 1.1% to 6.0%) and 3.0% (95% CI 0.6% to 5.4%), respectively, from 1997 to 2016. Suicide by poisoning increased significantly by 3.9% (95% CI 1.8% to 6.0%) in men from 1997 to 2016. In female suicides, poisoning increased by an average annual percentage change of 6.7% (95% CI 2.3% to 11.3%) over the 20-year period, showing a particularly sharp increase of 17% (95% CI 7.1% to 28.0%) per year from 1997 to 2005, before stabilising. Firearm use in men showed an annual decrease by 1.2% from 1997 to 2016, but it was not significant (95% CI 4.0% to 1.6%). Trends for firearm use in women could not be assessed as there were no reported deaths by firearm use in 2008.

**Figure 7:**
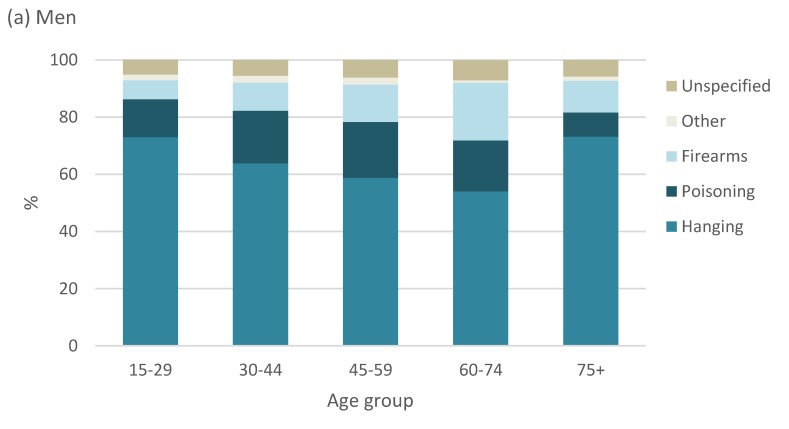

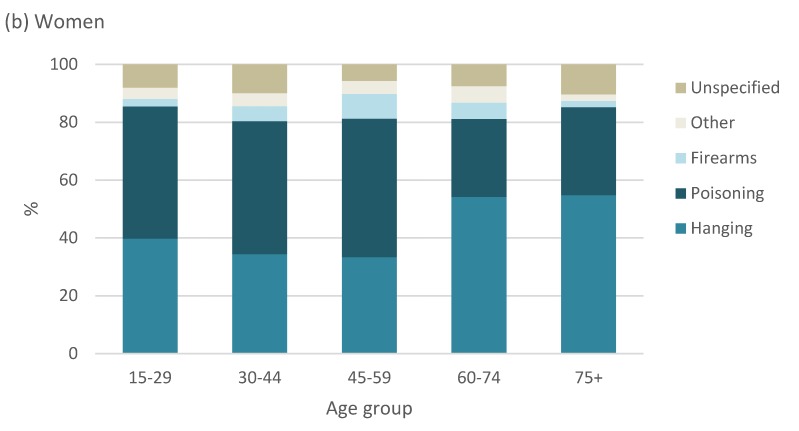
Distribution of suicides by method for broad age groups for men and women, South Africa, 1997–2016. (**a**) Men; (**b**) Women.

The distribution of the methods of suicide by broad age groups is shown in Figure 7. Hanging was the most common method of suicide in men across all age groups over the entire observation period and was more evident in young men aged 15 to 29 years and older men, 75 years and older. The use of firearms was most prevalent among men aged 60 to 74 years and in women aged 45 to 49 years. Poisoning was the most common method of suicide among younger women of 15 to 59 years, whereas older women of 60 years and older died by hanging. 

Table 2 describes the method of suicide by occupation group. Of the 877 (10.2%) suicides who had specified an occupation, hanging was a common method used by elementary workers (41.3%), service workers and armed forces (12.7%) and plant and machine operators (12.5 %). Firearm use was prevalent among service workers and armed forces (34.8%), skilled agricultural workers (13.9%) and managers (10.4%). Suicide by pesticide poisoning was a method most used by elementary workers, a large group that includes farm labourers (38.5%), and skilled agricultural workers (26.9%).

### 3.5. Years of Potential Life Lost

In South Africa, the burden of premature mortality was estimated as a total of 37,310,985 YPLL for all causes of death from 1997 to 2016, and less than 1% (0.65%, 243,429 YPLL) were due to suicide (Table 3). The average annual loss due to suicide was 9559 YPLL (rate, 5.73 per 10,000 population) among men and 2612 YPLL (rate, 1.49 per 10,000 population) among women. YPLL rates were highest in 2004 and 2005 for men, and in 2004 and 2014 for women. 

## 4. Discussion

This study provides a profile of suicide mortality in South Africa over a 20-year period from 1997 to 2016. We report a male predominance in suicide rates that is consistent with previous findings in South Africa [7,11,24] and international trends, with a few exceptions such as India [25] and China [26]. Joinpoint regression analyses indicate that the overall trend in suicide mortality changed twice for men and women in the observation period. Suicide mortality rates in women significantly increased by 12.6% from 1997 to 2004 and then stabilised; mortality rates in men remained stable over the observation period, despite the fluctuations across the years. 

We did not observe an overall decline in suicide mortality as reported by 2016 the Global Burden of Disease Study, where the largest significant decreases in mortality were observed in China, Denmark, the Philippines, Singapore and Switzerland [1]. Naghavi and colleagues reported increases in suicide mortality rates for LMICs such as Zimbabwe, and Zambia [1]. We observed wide year-on-year fluctuations in overall YPLL due to suicide but significant increases in the mortality rate of suicide by hanging for both sexes, while suicide by poisoning increased in men across the 20 years. Our results are consistent with findings from South Korea [27] and Norway [28], where increases in suicide rates by hanging for men and women have also been reported. Hanging was the most frequently used suicide method in South Africa, accounting for 47% of all suicide deaths, in keeping with previous South African studies [7,11]. A possible explanation for the increase in suicide by hanging may have been due to a substitution of other means [29], possibly due to the decrease in suicide by firearm use. During the study period, stricter gun control was applied in South Africa through the Firearms Control Act (FCA) of 2000; thus, stringent licencing conditions may have reduced access to firearms. Traditionally, women have chosen less lethal suicide methods such as poisoning while men chose highly lethal methods with such as hanging and firearm use [30]. In our study, men clearly chose hanging over other methods and women were as likely to die by hanging before 2000 and after 2015. This is of particular concern, as hanging has a high fatality rate of more than 70% [31]. Furthermore, it presents a challenge with respect to prevention efforts, as hanging is often chosen for its easy access to everyday household items such as ropes and belts that can be used as ligatures [32]. 

Notable changes in suicide by poisoning were shown in men and women. We report an increase of nearly 4% in suicide poisonings in men annually, while poisoning in women had stabilised after 2004. The dominant method of poisoning was with drugs and was more prevalent among women than men. This finding is similar to an earlier study by Eddleston (2000) who reported that medicines constituted more than 70% of all suicide by poisoning in sub-Saharan Africa [33] and was more recently confirmed by a poison centre in Cape Town, South Africa [34]. Further research is therefore needed to establish whether the restriction of over the counter medication may assist in reducing access to lethal means. Pesticide poisoning accounts for 30% of all suicides in the world [35] and remains an important method of suicide in sub-Saharan Africa [5]. However, the prevalence of pesticide poisoning in our study was considerably lower than reported in Ethiopia [36] and Uganda [37] but was in keeping with findings from South African mortuary studies in Bloemfontein [12] and Cape Town [38]. 

Suicide mortality is often linked to easy occupational access to a specific method of suicide [10]. Our study reports increased odds of dying by suicide among elementary workers, service workers and armed forces and agricultural workers compared to technicians. Similar to our findings, occupations associated with the use of or access to firearms such as armed forces and policemen have reported higher levels of firearm suicides [39]. Higher socioeconomic occupations, such as managers, may also suggest easier access to firearms. The association between suicide by pesticide poisoning and agricultural occupations have been clearly described [40], and farmers, farmworkers and agricultural populations with access to pesticides are at increased risk of suicide [41]. Studies have shown that reducing the accessibility of suicide means have been associated with a reduction in suicide rates [29]. Thus, there is a need for future studies to investigate explanations for the observed differences across occupations in South Africa. In addition, we also reported an increased odds of suicide among unemployed individuals. Unemployment is an independent risk factor of suicide as it is associated with an increased risk of mental illness [42], vulnerability to stressful life conditions and often, can contribute towards a lack of social cohesion [43]. The highest suicide rates were observed among men in 2005. Among the multitude of social and economic determinants of suicide, the rising levels of unemployment in South Africa could partly explain why men, especially of working age, were particularly affected [44].

When analysing mortality rates by sex and age, the suicide rates were highest in men, aged 15 to 44 years, and women older than 75 years. These results have been confirmed by a forensic investigation by Matzopoulus et al. [6] and were similar to international studies [45]. Southern sub-Saharan Africa’s age-standardised rate of years of life lost from suicide was estimated at 664.1 per 100 000 in 2016 (men, 1056.6 YLL per 100,000 and women, 296.1 YLL per 100,000) [1]. We report a lower average annual loss due to suicide for men (9559 YPLL, rate, 57.3 per 100,000 population) and women (2612 YPLL, rate, 14.9 per 100,000 population) in South Africa, suggesting a relatively lower suicide rate adjusted for age and sex compared to southern sub-Saharan Africa. Nevertheless, the risk of suicide in young South African men of working age has economic implications and should be investigated further, especially in occupations, where the prevalence of suicide is high. Consequently, as suicide is more common among the young and the elderly in South Africa, both YPLL and suicide rates should be used for monitoring mental health programs in order to reduce premature mortality due to suicide.

Several reviews have linked suicide with social inequalities [42,46]. In low- and middle-income countries, completed suicide has been associated with poverty [47], unemployment [48] and debt [49] and low education levels [50]. We found that the risk of suicide increased with educational levels. This was comparable to an Italian study where individuals with higher educational attainment were at greater risk of suicide [51]. Being married was associated with an increased risk of suicide compared to widows or divorced. Interpersonal difficulties such as marriage problems, family conflict and domestic violence may play an important role [52]. We report seasonal variation of suicide among adults, with a higher prevalence of suicide reported in summer and lowest in winter. This finding is corroborated with an earlier study by Flischer et al. who suggested that changes in social activities and the possible influence of Christmas may increase the risk of suicide, especially among those living in less urban areas and with lower socioeconomic status [53]. 

### Strengths and Limitations

This study has a few strengths. Firstly, this study consists of the analysis of a large national dataset and assessed changes in suicide mortality rates using joinpoint regression analyses. Joinpoint regression analysis calculates the annual percentage change in the mortality rates and can be used to study trends in suicide mortality over time. Secondly, this study provides estimates of years of potential life lost, which weighs death more heavily at a younger age than older individuals. Therefore, it provides additional information about the burden of premature death due to suicide. 

Our findings are derived from data in the Statistics South Africa database, which are obtained from death certificates. The quality of the death certificates is the main limiting factor, and underreporting of suicide have been previously described in the vital registration data, which can result in an underestimation of the true mortality burden attributable to suicide in South Africa [6,54]. Ajdacic-Gross et al. also observed that less violent methods such as suicide by poisoning and drowning are more likely to be underreported compared to highly lethal methods such as hanging and firearm suicides [5,9]. Thus, caution is needed when interpreting trends, especially suicide, by poisoning. It was difficult to accurately assess occupations at an increased risk of suicide due to the large proportion of occupations that were not specified. However, it is unlikely that systematic misclassification bias would have affected deaths by suicide. A further consideration was that we did not adjust or correct for the wrong assignment or ill-defined causes of death. In the study period, 11.6% (1,196,031) of deaths were assigned as ill-defined or unknown causes of death (R99). Redistribution of ill-defined causes may result in more reliable estimates of suicide mortality rates. 

## 5. Conclusions

Suicide mortality and method of suicide vary by sex and between age groups in the South African population across 20 years. Completeness of death registration data is necessary for accurate calculation of mortality rates. While the quality of death registration data has improved [14,54], ongoing training is needed on death certification skills. Efforts to improve our understanding of the epidemiology of suicide should continue in order to reduce the morbidity and mortality associated with suicide. Surveillance of vital registry data is a critical epidemiological tool for evaluating the effectiveness of health services and current mental health policies and can inform targeted prevention strategies for those at increased risk of suicide in South Africa.

## Figures and Tables

**Table 1:** Crude and adjusted suicide mortality odds ratios (MOR) of socio-demographic factors associated with suicide mortality in adults, 15 years and older (n = 8573).

Characteristic	n (%)	Crude MOR ^a^	95% CI	Adjusted MOR ^a^	95% CI	*p*-Value
Years						
1997–2001	1405 (16.4)	Reference		Reference		
2002–2006	2318 (27.1)	1.12	1.05–1.20	1.03	0.93–1.14	0.571
2007–2011	2142 (24.9)	1.03	0.96–1.10	1.07	0.96–1.18	0.215
2012–2016	2708 (31.6)	1.57	1.47–1.67	1.67	1.15–1.85	<0.001
Sex						
Female	1855 (21.9)	Reference		Reference		
Male	6699 (78.1)	3.33	3.16–3.50	2.68	2.49–2.89	<0.001
Broad age-group						
15–29	3656 (42.7)	17.57	15.99–19.28	8.42	7.31–9.70	<0.001
30–44	2581 (30.1)	6.50	5.90–7.16	3.79	3.29–4.33	<0.001
45–59	1301 (15.2)	4.02	3.62–4.45	2.19	1.91–2.53	<0.001
60–74	536 (6.3)	1.75	1.54–1.98	0.84	0.69–1.01	0.065
75 and over	499 (5.82)	Reference				
Population group						
Black African	5594 (65.3)	Reference		Reference		
Indian or Asian	156 (1.8)	1.24	1.15–1.35	1.36	1.13–1.64	0.001
White	763 (8.9)	1.25	1.07–1.47	2.52	1.13–1.64	<0.001
Coloured	958 (11.2)	2.12	1.99–2.27	1.64	1.45–1.84	<0.001
Unspecified or unknown	1102 (12.8)	0.48	0.44–0.51	0.66	0.59–0.73	<0.001
Marital status						
Widowed/divorced	531 (6.2)	Reference		Reference		
Never married	5739 (67.2)	2.88	2.64–3.16	1.02	0.90–1.17	0.670
Married	1610 (18.8)	1.77	1.60–1.96	1.13	0.99–1.29	0.065
Educational attainment						
None/Grade R	420 (8.7)	Reference		Reference		
Primary education	1221 (25.1)	1.83	1.63–2.04	1.13	1.02–1.28	0.024
Secondary	2897 (59.7)	3.23	2.91–3.58	1.49	1.34–1.67	<0.001
Tertiary	313 (6.5)	3.46	2.99–4.00	1.63	1.39–1.91	<0.001
Smoking status of deceased						
No	2150 (25.1)	Reference		Reference		
Yes	1966 (22.9)	2.05	1.93–2.19	1.24	1.15–1.32	<0.001
Unknown/unspecified	4457(51.9)	1.02	0.97–1.07	1.37	0.73–2.55	0.321
Province of death						
Free State	489 (5.7)	Reference		Reference		
Kwa-Zulu Natal	2690 (31.5)	1.99	1.81–2.19	1.98	1.77–2.21	<0.001
Western Cape	1094 (12.8)	2.02	1.82–2.25	1.10	0.93–1.31	0.252
Eastern Cape	972 (11.4)	1.09	0.98–1.22	1.00	0.88–1.16	0.899
Northern Cape	932 (10.9)	5.70	5.11–6.36	5.15	4.48–5.91	<0.001
Mpumalanga	683 (8.0)	1.51	1.34–1.69	1.00	0.86–1.16	0.987
North West	612 (7.2)	1.07	1.21–1.53	1.37	1.18–1.61	<0.001
Limpopo	571 (6.7)	1.31	0.95–1.21	1.00	0.87–1.17	0.939
Gauteng	482 (5.7)	0.39	0.34–0.44	0.34	0.28–0.39	<0.001
Occupation groups						
Technicians	25 (0.3)	Reference		Reference		
Managers	36 (0.4)	1.37	0.82–2.82	1.54	0.86–3.06	0.138
Professionals	70 (0.8)	1.03	0.65–1.63	1.63	0.97–2.73	0.064
Clerks	36 (0.4)	1.09	0.65–1.81	1.69	0.57–1.81	0.338
Service workers and armed forces	139 (1.6)	1.85	1.21–2.84	1.88	1.16–3.06	0.011
Skilled agricultural workers	92 (1.1)	2.62	1.68–4.09	2.72	1.64–4.51	<0.001
Craft & related trade workers	101 (1.2)	1.24	0.80–1.92	1.26	0.76–2.08	0.361
Plant & machine operators	107 (1.3)	1.40	0.91–2.18	1.52	0.61–1.91	0.096
Elementary occupations	332 (3.9)	1.25	0.83–1.88	1.69	1.06–2.71	0.027
Unspecified occupations or not economically active	7635 (89.1)	0.90	0.61–1.33	1.94	1.23–3.06	0.004
Place of death						
Hospital	1628 (19.0)	Reference		Reference		
Home	2301 (26.8)	2.21	2.08–2.36	2.26	2.08–2.46	<0.001
Dead on arrival	597 (7.0)	6.94	6.31–7.62	3.84	3.40–4.31	<0.001
Emergency room	165 (1.9)	2.62	2.23–3.08	1.84	1.48–2.27	<0.001
Nursing home	77 (0.9)	0.96	0.76–1.21	1.21	0.87–1.67	0.256
Unspecified	3805 (44.4)	4.34	4.09–4.60	3.37	3.11–3.66	<0.001

^a^ MOR—mortality odds ratio.

**Table 2:** Methods of suicide by occupation group (n = 877).

Occupation Group	Hanging	Firearms	Pesticide Poisoning	Poisoning by other Means	Unspecified or Other Means
Managers	8 (1.6)	12 (10.4)	0 (0)	8 (4.8)	2 (3.8)
Professionals	32 (6.2)	10 (8.7)	1 (3.9)	15 (8.9)	6 (11.3)
Technicians	14 (2.7)	1 (0.8)	0 (0)	7 (4.2)	2 (3.8)
Clerks	15 (2.9)	7 (6.1)	1 (3.9)	8 (4.8)	3 (5.7)
Service workers and armed forces	65 (12.6)	40 (34.8)	2 (7.7)	24 (14.4)	4 (7.5)
Skilled agricultural workers	53 (10.3)	16 (13.9)	7 (26.9)	11 (6.6)	3 (5.7)
Craft and trade workers	51 (9.9)	8 (6.9)	2 (7.7)	23 (13.7)	10 (18.9)
Plant and machine operators	64 (12.5)	11 (9.6)	3 (11.5)	17 (10.2)	2 (3.8)
Elementary occupation	212 (41.3)	10 (8.7)	10 (38.5)	54 (32.3)	23 (43.4)

**Table 3:** Years of potential life lost (YPLL) estimates by all causes of death and suicide-specific cause of death by year and sex.

Year	Male				Female			
	All Causes (YPLL)		Suicide-Specific (YPLL)		All Causes (YPLL)		Suicide-Specific (YPLL)	
	Total YPLL	Rate Per	Total PYLL	Rate Per	Total PYLL	Rate Per	Total PYLL	Rate Per
		1000		1000 ^a^		1000 ^a^		1000 ^a^
1997	2,398,233	209,88	2325	0.20	1,471,662	116.12	595	0.04
1998	2,792,467	244,48	3912	0.29	1,862,922	145.78	1237	0.09
1999	2,997,012	262,39	6595	0.51	2,223,997	172.00	1805	0.13
2000	3,263,040	286,10	6625	0.52	2,694,757	207.61	2035	0.14
2001	3,598,960	274,09	11,635	0.78	3,135,470	207.78	2712	0.17
2002	3,988,685	296,95	6297	0.42	3,737,317	248.45	1667	0.09
2003	4,421,255	323,76	6967	0.45	4,295,517	281.71	2007	0.12
2004	4,555,435	328,59	11,152	0.72	4,638,935	300.71	3330	0.20
2005	4,653,162	330,58	13,457	0.85	4,725,847	302.97	3322	0.19
2006	4,717,585	328,82	13,370	0.81	4,707,187	297.83	2712	0.16
2007	4,671,250	319,02	10,507	0.62	4,483,875	279.87	3237	0.18
2008	4,580,495	306,39	10,565	0.62	4,307,242	264.37	2920	0.16
2009	4,386,215	276,70	8305	0.47	4,725,847	282.27	2885	0.16
2010	4,058,652	260,99	8822	0.50	3,661,447	216.78	2852	0.15
2011	3,705,815	233,27	8767	0.48	3,164,912	184.45	2422	0.13
2012	3,505,240	214,99	11,500	0.60	2,860,962	163.65	3532	0.18
2013	3,356,222	201,23	14,272	0.75	2,605,087	146.71	3265	0.17
2014	3,284,210	192,78	14,370	0.75	2,457,035	136.29	4065	0.21
2015	3,249,292	186,68	11,252	0.58	2,331,967	127.35	3227	0.17
2016	3,133,707	175,98	10,485	0.53	2,187,140	117.53	2422	0.13
**Total**	28,015,087	262,68	191,180	0.57 ^b^	9,295,898	210.01 ^b^	52,249	0.15 ^b^

^a^ Rate per 1000—age-standardised rate per 1000 population. ^b^ Average annual YPLL rate per 1000 population.
